# Genome-Wide Identification and Characterization of the *Pirin* Gene Family in *Nicotiana benthamiana*

**DOI:** 10.3390/genes16020121

**Published:** 2025-01-22

**Authors:** Gecheng Xu, Jingjing Shi, Jiliang Qiao, Pingan Liao, Bin Yong, Kaili Zhong

**Affiliations:** 1 State Key Laboratory for Managing Biotic and Chemical Threats to the Quality and Safety of Agro-Products, Key Laboratory of Biotechnology in Plant Protection of MARA, Key Laboratory of Green Plant Protection of Zhejiang Province, Institute of Plant Virology, Ningbo University, Ningbo 315211, China; xxxgccc@163.com (G.X.); 18958360656@163.com (J.S.); yongbinforage@163.com (B.Y.); 2 Luohe Academy of Agricultural Sciences, No. 900, Huanghe Road, Luohe 462300, China; qjl8835@163.com (J.Q.); liaopingan@126.com (P.L.)

**Keywords:** Pirin proteins, abiotic stress, biotic stress, viral infections, *Nicotiana benthamiana*

## Abstract

**Background**: Pirins are nuclear cupin proteins, one of several gene families within the plant cupin superfamily. However, the identification and functional analysis of Pirin proteins in *Nicotiana benthamiana* have not been explored. **Methods**: In this study, genome-wide analysis identifying NbPirin genes in *N. benthamiana* was conducted, as was phylogenetic analysis of Pirin genes in four Solanaceae species (including *Capsicum annuum*, *Solanum lycopersicum*, *Solanum tuberosum*, and *N. benthamiana*). In addition, we also evaluated the expression pattern of NbPirins under abiotic stress (temperature and phytohormones) and biotic stress (TMV, TuMV, and PVX). **Results**: A total of six Nbpirin genes were identified, which can be divided into three clades, and NbPirins also embraced a variety of abiotic or biotic cis-acting elements. The results showed that the expression of NbPirin1-6 was influenced by temperature variations, of which NbPirin6 was significantly upregulated at high temperatures (42 °C) but downregulated at low temperatures (4 °C). Notably, the expression of NbPirin6 exhibited a consistent decrease under ABA and MeJA treatments. Moreover, the expression of NbPirin1-6 was also affected by TMV, TuMV, and PVX infection. NbPirin1, NbPirin2, NbPirin3, and NbPirin5 showed higher expression levels under different viral infections compared to non-infection. Interestingly, NbPirin3 showed the highest expression level during TuMV infection (approximately a 20-fold increase compared to non-infection). **Conclusions**: Our study proposes the potential role of NbPirin6 in plant responses to abiotic stress, and the role of NbPirin3 in plant antiviral defense, and further lays the groundwork for future research on the functions of NbPirin proteins in responses to various stressors.

## 1. Introduction

Gene duplication events, which lead to the formation of multigene superfamilies, constitute a key mechanism by which plants adapt and survive repeated exposure to biotic and abiotic stressors. Among these superfamilies, the cupin superfamily is a highly versatile gene family in plants [1]. Cupin superfamily proteins are characterized by conserved β-barrel structures and unique metal ion-binding sequences. The name “cupin” is derived from the β-barrel structure in their protein sequences, with “cupa” in Latin meaning a small barrel [2]. This family is defined via a conserved amino acid sequence (HI/THPRATEI) [1]. As members of the cupin superfamily, Pirins have a characteristic bicupin fold and can bind iron ions [3]. Pirin proteins are highly conserved across diverse biological kingdoms, including bacteria, fungi, plants, and mammals, suggesting their essential role in biological evolution [4]. This family includes eukaryotic and prokaryotic Pirin proteins. The gene encoding this protein is expressed in all body tissues, with the highest expression levels found in the liver and heart. Pirin is a nuclear protein that is localized exclusively in the cytoplasm and is predominantly concentrated in punctate subnuclear structures [3,5]. This *Pirin* gene family is relatively underexplored in plants, which is one of the reasons this study was conducted. Its sequence similarity to human *Pirin* led to the finding of *At-Pirin1* in *Arabidopsis thaliana*. There are four members (*At-Pirin1 to At-Pirin4*) of the *Pirin* family in *A. thaliana*, all of which contain two cupin motifs. However, *At-Pirin3* and *At-Pirin4* have a slightly shorter motif than the other two members (motif 1) [6]. Reports found members of the *Pirin* gene family in wheat, a hexaploid species with three genomes (A, B, and D). Researchers have identified 18 *Pirin* genes in wheat, which can be classified into six categories (*Ta-Pirin-1* to *Ta-Pirin-6*). Each diploid genome contains six homologous genes, with one copy in each subgenome (A, B, and D), referred to as homologues from different genomes [7].

*Pirins* have diverse functions in various organisms. In humans, the Pirin protein was first discovered through its interplay with nuclear factor I and has been shown to function as a transcriptional co-regulator of several transcription factors [5,8,9]. Pirins have also been studied in bacteria, where they play a role in the biosynthesis of antibiotics. For example, in *Saccharopolyspora spinosa*, the deactivation of the *ssPirin* gene causes severe growth problems and hydrogen peroxide accumulation. The subsequent overexpression and knockout of *ssPirin* increases glucose consumption and utilization, attenuates the tricarboxylic acid (TCA) cycle, slows sporulation, and increases spore production. Moreover, the overexpression of *ssPirin* in *S. spinosa* promotes the β-oxidation pathway and increases spinosad production. However, the knockout of *ssPirin* almost completely abolishes spinosad production [10]. These results suggest that *Pirin* plays a crucial role in the production of antibiotics. In Escherichia coli, the YhaK protein, a Pirin homologue, is significantly upregulated under oxidative stress and highly expressed during biofilm growth [11].

In plants, Pirin proteins have various functions. In *A. thaliana*, Pirin proteins regulate quercetin levels, which influence plant responses to specific light and UV conditions. This regulation is critical during the transition from seeds to seedlings, a key stage in plant growth and development [12]. Heterotrimeric G-proteins are involved across a range of signaling activities in plants. In *A. thaliana*, in vitro binding assays demonstrated that Pirin interacts with the G-protein α-subunit (GPA1). Further analysis of two Pirin T-DNA insertion mutants revealed phenotypes resembling those of GPA1 mutants, such as decreased germination in the absence of stratification, delayed germination, as well as early seedling development under abscisic acid (ABA) treatment. These results suggest that Pirin functions downstream of GPA1 to regulate seed germination and early seedling development [6]. These findings point to multiple roles for *Pirin* in early plant growth and development. Additionally, in *A. thaliana*, *Pirin* is involved in lignification. By modulating the expression of genes involved in lignin biosynthesis, this molecule suppresses the accumulation of S-type lignin near xylem vessels and promotes the enrichment of G-type lignin in the secondary cell walls of vessel elements [13]. Research has also shown that Pirin proteins are involved in how plants respond to pathogens. For example, *TvPirin*, a PRN gene that has been identified in the parasitic plant *Triphysaria versicolor*, was shown to be significantly upregulated in roots by the haustorium-inducing substance 2,6-dimethoxybenzoquinone [14]. In tomatoes, studies have indicated that camptothecin-induced programmed cell death (PCD) significantly increases LePirin transcription [15]. In barley, Pirin transcription is induced by pathogen-derived moniliformin, which triggers plant cell death [16,17]. *A. thaliana* PIRIN2 has been associated with susceptibility to the bacterial pathogen *Ralstonia solanacearum*. Mutants of the two alleles of the PIRIN2 gene demonstrated enhanced resistance to *R. solanacearum* infection. Further studies revealed that PIRIN2 interacts with the papain-like cysteine protease (PLCP) XCP2, thereby preventing its degradation. This stabilization of XCP2 increases susceptibility, suggesting that the PIRIN2-mediated stabilization of XCP2 underlies susceptibility to R. solanacearum [18]. To summarize, these studies demonstrate that Pirin proteins play complex roles in plant responses to various pathogens, with different Pirin proteins potentially exhibiting opposing functions.

*N. benthamiana* is a widely utilized experimental model in the field of plant biology. It has been cultivated and employed in numerous laboratories since its initial use in virus-related studies in the 1940s [19,20]. This model exhibits high susceptibility to infection by the tobacco mosaic virus (TMV), turnip mosaic virus (TuMV), and potato virus X (PVX) [21,22,23], and it responds in a variety of ways to abiotic stresses from the outside world [19,20,21,22], which are the focus of our study. Moreover, these aspects of the *Pirin* family in *N. benthamiana* are very scarcely studied. Therefore, *N. benthamiana* was selected as the subject of this experiment.

In this study, we analyzed the *N. benthamiana Pirin* (*NbPirin*) gene family and classified six *NbPirin* genes into three major groups based on their phylogenetic distribution and domain characteristics. Additionally, we performed a detailed analysis of their gene structures, chromosomal locations, domains, and *cis*-acting elements. We examined the expression profiles of six *NbPirins* in *N. benthamiana* tissues (roots, stems, and leaves) under abiotic stress (temperature and phytohormones) and biotic stress (TMV, TuMV, and PVX) using quantitative real-time PCR (qRT-PCR). We found that *NbPirin6* may play a major role in the plant’s response to external abiotic stress, while *NbPirin3* may be crucial in the plant’s response to external biotic stress. This study offers a valuable foundation for understanding the role of *NbPirins* in the stress response of *N. benthamiana*.

## 2. Materials and Methods

### 2.1. Identification of Pirin Genes in N. benthamiana

To identify potential *Pirin* genes in *N. benthamiana* (*NbPirins*), the *Triticum aestivum Ta-Pirin-1A* (TraesCS4A02G336200.1) amino acid sequence was used to search the hidden Markov model (HMM) [7]. TBtools software (Version 2.136) was employed to search the *N. benthamiana* database (Nicotiana_benthamianaV261) available on the Sol Genomics Network (https://solgenomics.net/, accessed on 21 October 2024). Additional *Pirin* genes from other Solanaceae species were identified using the same approach. The *Capsicum annuum* database (C.annuum_Dempsey.v1.0) was also downloaded from the Sol Genomics Network, and the *Solanum lycopersicum* database (Solanum lycopersicum ITAG5.0) and *Solanum tuberosum* database (Solanum tuberosum v6.1) were downloaded from Phytozome (https://phytozome-next.jgi.doe.gov/, accessed on 21 October 2024) for further analysis. The members of the *NbPirin* gene family were checked using Pfam (https://www.ebi.ac.uk/interpro/search/sequence/, accessed on 21 October 2024) and the NbPirin protein sequences were also obtained.

### 2.2. Characterization of NbPirins

Information about the *NbPirin* gene family was successfully retrieved, including their chromosomal locations, the lengths of their coding DNA sequences (CDS), and the number of amino acids they encode. This data was obtained from Ensembl Plants (http://plants.ensembl.org/, accessed on 21 October 2024). Additionally, the theoretical isoelectric point (pI) and molecular weight (MW) of each NbPirin protein were determined using ExPASy (https://web.expasy.org/compute_pi/, accessed on 21 October 2024).

### 2.3. Multiple Sequence Alignments and Phylogenetic Analysis

Four data sets of six identified NbPirin protein sequences, two Pirin protein sequences in *C. annuum* (*CaPirins*), four Pirin protein sequences in *S. lycopersicum* (*SlPirins*), and four Pirin protein sequences in *S. tuberosum* (*StPirins*) were used for phylogenetic analysis. Multiple sequence alignments were performed using MEGA-11 software (version 11.0.13) with the MUSCLE function. A phylogenetic tree was constructed using the neighbor-joining method, and bootstrap analysis was conducted with 1000 replicates to assess the reliability of the results [19].

### 2.4. Gene Structural Domain, Gene Structure, and Motif Analysis of NbPirin

The NbPirin protein sequences were submitted to the NCBI Batch CD-Search tool (https://www.ncbi.nlm.nih.gov/Structure/bwrpsb/bwrpsb.cgi, accessed on 23 October 2024), and TBtools software was utilized to obtain and visualize gene structural domain data. Gene annotation files for *N. benthamiana* were obtained from the Sol Genomics Network (https://solgenomics.net/, accessed on 21 October 2024), and gene structures were analyzed using TBtools Gene Structure View (Version 2.136). The prediction of the motifs of *NbPirin* was conducted by means of the MEME Suite Network (https://meme-suite.org/meme/tools/meme, accessed on 23 October 2024), an online analysis tool. This analysis identified ten conserved motifs.

### 2.5. Analysis of Chromosomal Location and Duplication

The *N. benthamiana* genomic sequences and genome annotation files were obtained via the Sol Genomics Network (https://solgenomics.net/, accesses on 21 October 2024). A graph depicting the chromosomal location and duplication relationships of *NbPirin* was generated using TBtools software (Version 2.136), One Step MCScanX (Version 2.136), and Dual Synteny Plot (Version 2.136) [21].

### 2.6. The Calculation of the Ka/Ks Values

When the Ka/Ks value exceeds 1, the mutations are advantageous under specific selective pressures. When the Ka/Ks value equals 1, the mutations can be considered to be neutral. Conversely, a Ka/Ks value less than 1 indicates that the mutations are subject to purifying selection. The calculation of Ka and Ks values was conducted utilizing the Ka/Ks Calculator function within the TBtools software suite. The divergence time (T) was calculated as T = Ks/(2 × 9.1 × 10^−9^) × 10^−6^ million years ago (Mya).

### 2.7. Cis-Acting Regulatory Elements Analysis

The promoter regions of the *NbPirin* gene, spanning two kilobases upstream of the transcription start site, were obtained from the *N. benthamiana* database. The utilization of these DNA sequences in the identification of *cis*-acting regulatory elements was achieved via the PlantCARE database (http://bioinformatics.psb.ugent.be/webtools/plantcare/html/, accesses on 26 October 2024).

### 2.8. Tissue-Specific Expression of NbPirin

The expression of all *NbPirin* genes was analyzed in three tissues of *N. benthamiana*: leaves, stems, and roots. Each tissue sample was collected in triplicate and stored at −80 °C until total RNA extraction and gene expression analysis using quantitative real-time PCR (qRT-PCR) were conducted. Tissue-specific expression results were visualized as histograms using GraphPad Prism 9.5.

### 2.9. Plant Growth, Abiotic Stress, and Biotic Stress Treatment

*N. benthamiana* seeds were cultivated within a growth chamber maintained at a constant temperature of 25 ± 2 °C and a relative humidity of 70%, using long-day conditions (16 h light/8 h dark cycles) [19]. Gene expression levels in relation to stress treatments were analyzed using the cultivated *N. benthamiana*. For temperature stress experiments, *N. benthamiana* were cultivated at 25 °C (control), 4 °C, and 42° C. For phytohormone stress experiments, *N. benthamiana* were treated with 100 μM abscisic acid (ABA) solution (purchased from Solarbio, Beijing, China, CAS Number: 14375-45-2), 100 μM methyl jasmonate (MeJA) solution (purchased from Sigma, Shanghai, China, CAS Number: 39924-52-2), or distilled water as a control [23,24]. All samples were obtained from the same leaf position of each *N. benthamiana* plant at five time points (0, 2, 4, 6, and 12 h), with three biological replicates for each sample.

To evaluate viral resistance in *N. benthamiana*, four-week-old plants were inoculated with inoculation buffer (control), TMV-GFP (GFP-labeled TMV), TUMV-GFP (GFP-labeled TUMV), or PVX. Viral transcription and frictional inoculation were carried out as described in the published literature [19]. Following inoculation, the plants were cultivated in a mixture of soil matrix (peat:vermiculite = 1:1) under long-day photoperiods at 25 ± 2 °C and 70% relative humidity. One week after the initial virus infestation, samples were obtained from the same leaf position of each plant, with a minimum of three biological replicates for each treatment.

Leaf samples were snap-frozen in liquid nitrogen immediately after collection and stored at −80 °C prior to total RNA extraction. Gene expression was determined using qRT-PCR. Each experimental condition included three biological replicates. The results were analyzed using GraphPad Prism 9.5 and presented as histograms.

### 2.10. RNA Extraction and Gene Expression Analysis by qRT-PCR

Total RNA was extracted using the HiPure Plant RNA Mini Kit (Magen) from each sample according to the manufacturer’s instructions. The samples were stored at −80 °C. First-strand cDNA was synthesized using the First Strand cDNA Synthesis Kit (Toyobo, Kita-ku, Osaka, Japan) as described by the manufacturer and in previous studies [25,26]. Quantitative real-time polymerase chain reaction (qRT-PCR) was performed using the ABI7900HT Sequence Detection System (Applied Biosystems QuantStudio 5, Foster City, CA, USA) and Hieff qPCR SYBR Green Master Mix (Yeasen, Shanghai, China) to validate the expression levels of *NbPirins*. The qRT-PCR conditions were as follows: 10 min at 95 °C, followed by 40 cycles of 15 s at 95 °C, 30 s at 60 °C, and 30 s at 72 °C. *N. benthamiana* ubiquin C (NbUBC) served as the internal reference gene. The complete list of primers utilized is provided in Table S4. The relative expression levels of *NbPirins* were calculated by means of the 2^−ΔΔCt^ method [27]. A minimum of three biological replicates and three technical replicates were used for each treatment. The results were analyzed using GraphPad Prism 9.5 and presented as histograms.

## 3. Results

### 3.1. Genome-Wide Identification and Characterization of the Pirin Gene Family in N. benthamiana

**Figure 1:**
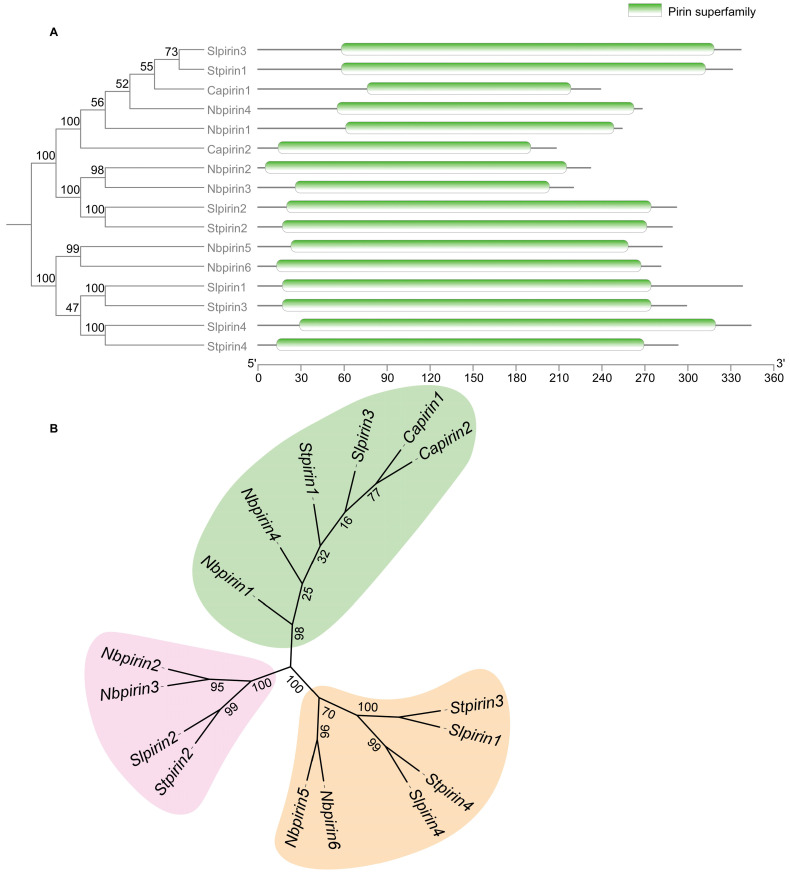
Phylogenetic tree based on alignment of Pirin proteins from *N. benthamiana*, *C. annuum*, *S. lycopersicum*, and *Solanum tuberosum*. (**A**) Phylogenetic relationships and conserved domains in NbPirin proteins. (**B**) Phylogenetic tree of Pirins from *S. tuberosum*, *S. lycopersicum*, and *C. annuum*. The phylogenetic tree was generated via the neighbour-joining method via MEGA-11 software with 1000 bootstrap replicates. All the Pirins are divided into 3 subclasses represented by specific colored backgrounds: *St*, *S. tuberosum. Sl*, *S. lycopersicum. Ca*, *C. annuum*.

After a genome-wide search of *Pirin* homologues according to the Ta-*Pirin-1A* genes in *T. aestivum* [7], we identified a total of six candidate *Pirin* genes in *N. benthamiana*. The six candidate Pirin protein sequences were subsequently submitted to the Pfam database, and all were found to contain the *Pirin* superfamily domain, indicating that they are members of the *Pirin* gene family (*NbPirins*) (Figure 1A). Detailed characteristics of the *NbPirins*, such as gene ID in the Sol Genomics Network, chromosomal location, Coding DNA Sequence (CDS) length, number of amino acids, and physicochemical parameters, are provided in Table S1. The CDS lengths ranged from 663 to 849 bp. The number of encoded amino acids was between 220 and 282 (aa). The largest protein was *NbPirin5* (Niben261Chr10g0844008.1) and the smallest protein was *NbPirin3* (Niben261Chr09g0018012.1). In terms of the number of amino acids encoded, the predicted MW of NbPirin proteins varied from 24,542.94 to 31,336.58 kDa, and the theoretical pI ranged from 5.9 to 6.26. Based on these results, individual proteins that are members of the *NbPirin* gene family have similar physicochemical properties among *N. benthamiana*.

To elucidate the evolutionary relationships of, and to classify, *Pirins* in Solanaceae, we constructed a phylogenetic tree based on six *NbPirins* (*N. benthamiana Pirins*), two *CaPirins* (*C*. *annuum Pirins*), four *SlPirins* (*S*. *lycopersicum Pirins*), and four *StPirins* (*S*. *tuberosum Pirins*) (Figure 1B). The 16 *Pirins* were classified into three groups based on the phylogenetic trees and sequence similarity of all Pirin proteins. This phylogenetic tree revealed that NbPirin proteins have a high degree of homology with CaPirin, SlPirin, and StPirin proteins, as shown in Figure 1. Protein domain analysis revealed that all *NbPirins*, *CaPirins*, *SlPirins*, and *StPirins* had only one identical *Pirin* superfamily domain (Figure 1), implying that they may have similar functional roles. In addition, we identified several closely related gene pairs in the same species, including *NbPirin2* and *NbPirin3*, suggesting a paralogous origin.

### 3.2. Determination of the Chromosomal Location and Duplication of NbPirins

**Figure 2:**
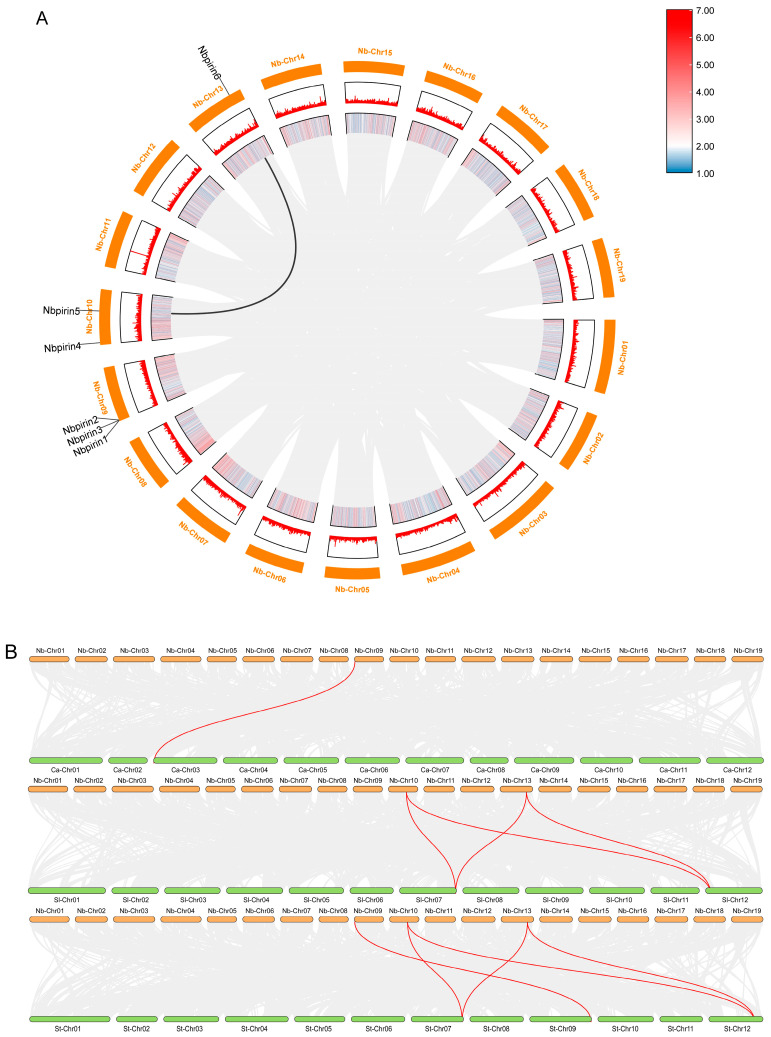
Duplication events of 6 *NbPirins* and syntenic analysis of *Pirin* genes between *N. benthamiana* and *C. annuum*, *S. lycopersicum*, and *S. tuberosum*. (**A**) Distribution and duplication events of the 6 *NbPirins*. Six *NbPirin* genes were mapped onto three *N. benthamiana* chromosomes. The chromosome number is given in the segment outside the outer circle. The inner line marks the only duplication event of different chromosomes. (**B**) Syntenic analysis of the *Pirin* genes among the four species.

To further investigate this, six *NbPirins* were mapped onto three chromosomes based on the genome annotation information (Table S1). Chromosomal locations were determined by TBtools, and the specific distribution of *NbPirins* is presented in Figure 2. There were one, two, and three *NbPirins* distributed on the Niben261Chr13, Niben261Chr10, and Niben261Chr09 chromosomes, respectively. Consequently, the chromosomal distribution of *NbPirins* exhibited a scattered and nonrandom pattern.

Two primary factors are instrumental in the augmentation of gene families in plants: tandem and segmental duplication [25]. From the chromosomal location map (Figure 2A), only one duplication of *NbPirins* was identified between Niben261Chr13 and Niben261Chr10, suggesting that these genes were paralogous.

To further clarify the evolutionary relationships among *NbPirins*, *CaPirins*, *SlPirins*, and *StPirins*, we generated syntenic maps for *N. benthamiana*, *S. tuberosum*, *S. lycopersicum*, and *C. annuum*. One, four, and six homologous *Pirin* gene pairs were identified in *N. benthamiana*, *C. annuum*, *S. lycopersicum*, and *S. tuberosum*, respectively (Figure 2B). These findings indicate a close evolutionary link between *N. benthamiana* and *S. tuberosum*.

### 3.3. Conserved Motif and Gene Structure Analyses

To elucidate the evolution of *NbPirin* family members, we performed MEME analysis, which identified 10 conserved motifs. *NbPirin5* and *NbPirin6* contain nine predicted motifs, except motif 6, whereas *NbPirin1* and *NbPirin3* possess 6 motifs. *NbPirin2* and *NbPirin4* contain 7 motifs, except motifs 6, 7, and 10, and motifs 3, 7, and 8, respectively (Figure S1). The gene structures of *NbPirins* are not conserved, and the lengths of intron sequences among different genes vary substantially, with the longest intron sequence found in *NbPirin1*. The exon and intron numbers and lengths of the *NbPirins* that cluster together are similar, with *NbPirin1* and *NbPirin4*, *NbPirin5*, and *NbPirin6* having the same number of exons and introns, whereas *NbPirin2* and *NbPirin3* have different numbers of exons and introns (Figure S1).

### 3.4. Evolutionary and Divergence Patterns

The Ka/Ks ratio is an indicator of selective pressure that acts on protein-encoding genes. Determining whether selective pressure is exerted on the protein-coding gene is contingent on the value of this ratio [26]. We calculated the Ka/Ks ratios of the *NbPirin5* and *NbPirin6* gene pair (Table S2). These finding revealed that the Ka/Ks ratio was 0.14167 (Table S2), and it was evident that these two genes were influenced mainly by high-purity selection [28]. The divergence time (T) was also calculated using the formula T = Ks/(2 × 9.1 × 10^−9^) × 10^−6^ Mya. The findings revealed that the differentiation time of the pair of pure (Nb-Nb) members was 6.25577 Mya (Table S2).

### 3.5. Prediction of Cis-Acting Regulatory Elements of NbPirins

**Figure 3:**
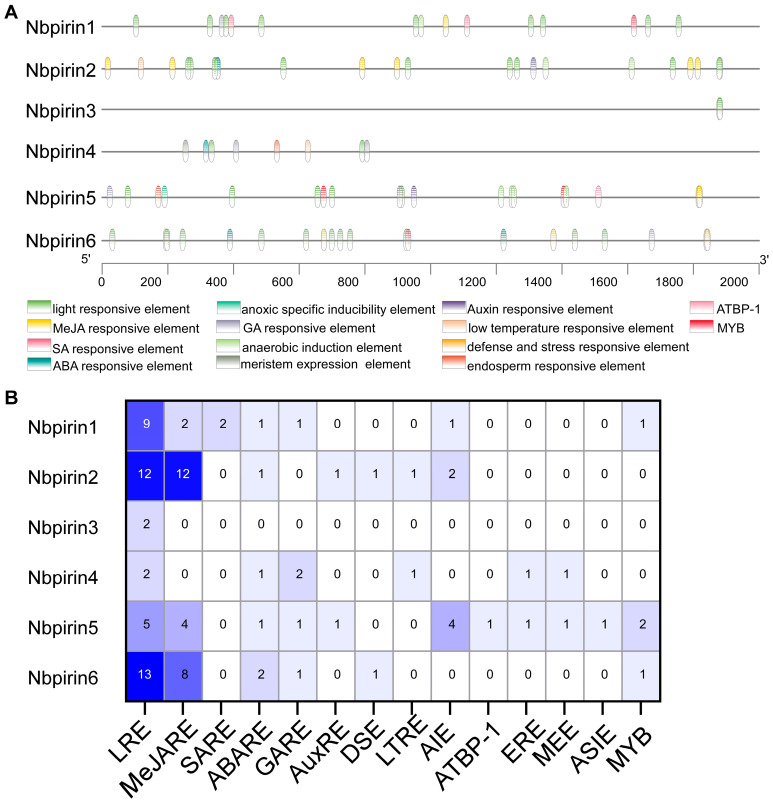
Prediction of *cis*-acting regulatory elements in *NbPirins*. (**A**) Names and positions of the *cis*-acting regulatory elements in the *NbPirins*. (**B**) The number of *cis*-acting regulatory elements detected in the promoter region of each *NbPirin*.

In plant biology, *cis*-acting regulatory elements can modulate gene transcription through binding with target transcription factors [29]. Many *cis*-acting elements have been identified in plants, with a significant proportion of these elements correlating with stresses originating from the external environment. These include abiotic stresses [30,31,32,33] and biotic stresses [34,35,36]. The predicted *cis*-acting regulatory elements of *NbPirins* are listed in Table S3. As shown in Figure 3, fourteen distinct elements were identified. The most abundant element was the light-responsive element (43 in total). Among the hormone-responsive elements, the most prominent was the methyl jasmonate (MeJA)-responsive element (26 in total). It has been established that there are six abscisic acid (ABA)-responsive elements, representing the second most common of all hormone-responsive elements. Furthermore, the presence of low-temperature-responsive, defense-, and stress-responsive elements was identified.

### 3.6. Tissue-Specific Expression of NbPirins

**Figure 4:**
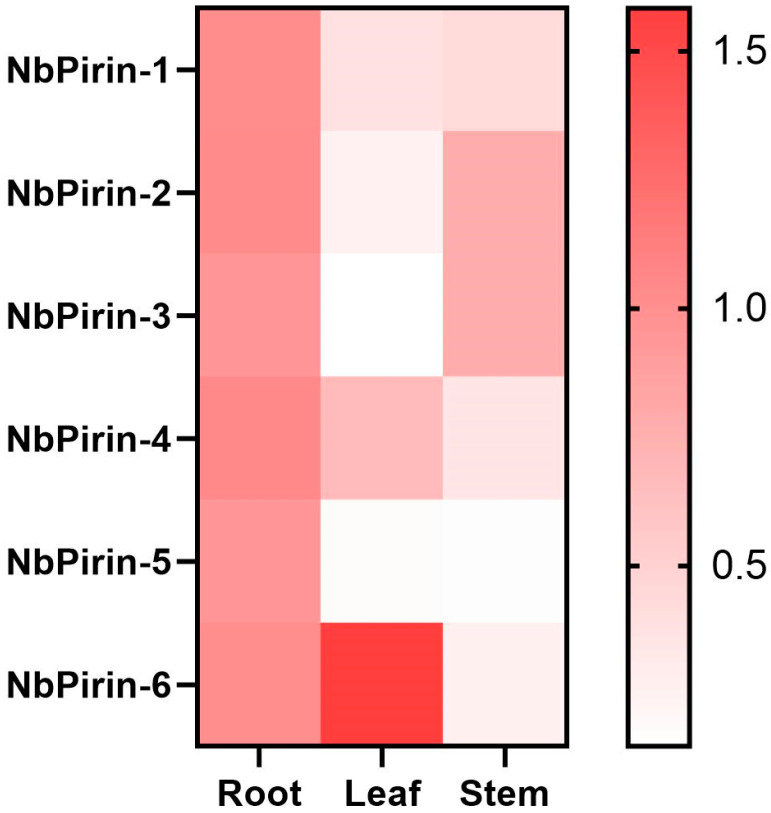
Differential expression of 6 *NbPirins* in the roots, leaves, and stems of *N. benthamiana* by qRT–PCR. The average expression of *NbPirins* in leaves and stems was calculated using the average expression of *NbPirins* at the roots as a reference. The color scale denotes the fold change (FC), with white to red signaling low to high expression levels.

Six *NbPirin* genes were chosen to investigate their expression in various tissues (roots, leaves, and stems) through qRT-PCR (Figure 4). The primers utilized are listed in Supplementary Table S4. As exhibited in Figure 4, relative to its expression level in the roots, the expression level of *NbPirin6* in the leaves was the highest among all the tissues, followed by NbPirin4. The expression levels of *NbPirin1* within leaves and stems were similar. Furthermore, we found that the expression levels of both *NbPirin2* and *NbPirin3* were decreased in the leaves, but their expression in the stems was the highest among all *NbPirins*. Notably, the expression of *NbPirin5* was very low within stems and leaves. These results showed that the expression patterns of *NbPirins* in *N. benthamiana* are not identical in different tissues.

### 3.7. Expression of NbPirins After Abiotic Stress Treatment

Analysis of the *cis*-acting regulatory elements revealed the presence of four distinct hormone-responsive elements (Figure 3). ABA-responsive elements (ABREs) respond to ABA, MeJA-responsive elements (MeJAREs) to MeJA, salicylic acid (SA)-responsive elements (SAREs) to SA, and gibberellin (GA)-responsive elements (GAEs) to GA. It was predicted that, of the hormone responsive elements identified, MeJAREs would be the most prevalent, followed by ABAREs. A total of 26 MeJAREs were identified within the hormone-responsive elements. In addition, we identified low-temperature-responsive elements (LTREs) and defense- and stress-responsive elements (DSEs).

**Figure 5:**
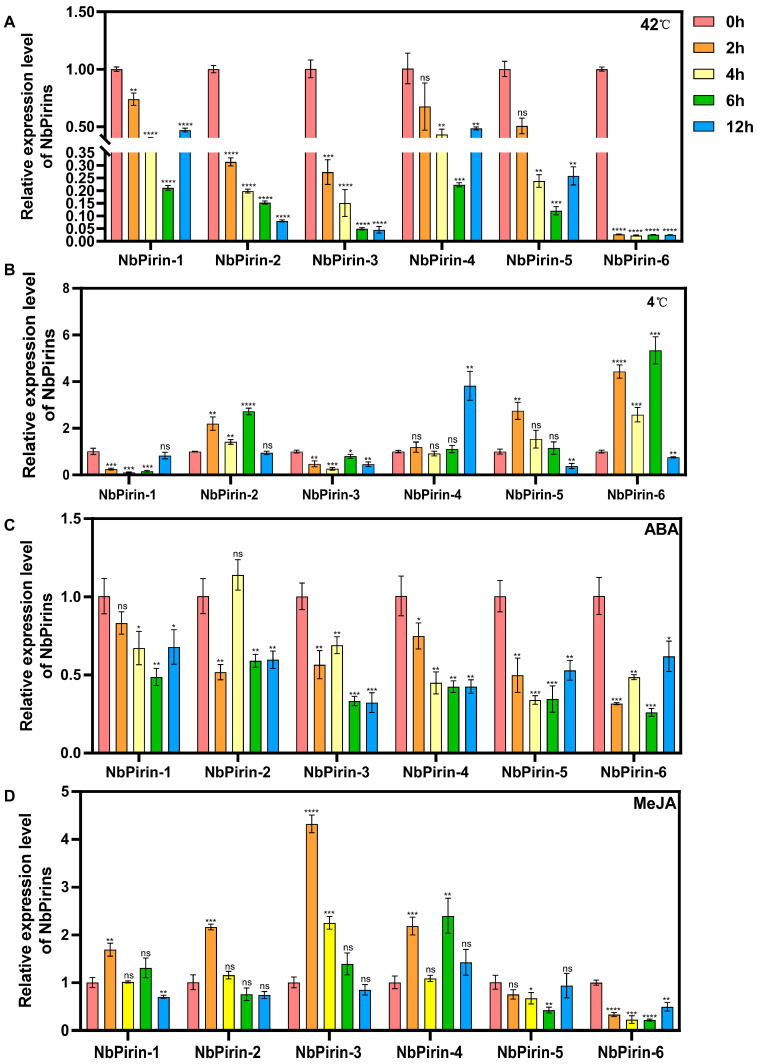
The relative expression of NbPirins after different abiotic stress treatments. (**A**) 42 °C treatment. (**B**) 4 °C treatment. (**C**) ABA treatment. (**D**) MeJA treatment. Three independent replicates were conducted. Data is the mean ± SD (Student’s *t*-test, ns = not significant, *n* = 3, * *p* < 0.05, ** *p* < 0.01, *** *p* < 0.001, **** *p* < 0.0001).

Hence, we explored the functions of the above-mentioned elements of *Pirin* genes. To evaluate their expression levels after different treatments, we selected six *NbPirins* as representative genes and performed qRT-PCR. *N. benthamiana* plants were exposed to high (42 °C) and low (4 °C) temperatures for 12 h, and RT–qPCR analysis was used to monitor changes in the *NbPirin* expression levels starting at 0 h. At high-temperature conditions (42 °C), we observed changes in *NbPirin* expression at five time points: 0 h, 2 h, 4 h, 6 h, and 12 h (Figure 5A). The expression levels of all the *NbPirins* decreased sharply after 0 h. *NbPirin6* expression exhibited the most significant and rapid reduction, reaching its lowest level by 2 h. *NbPirin2* and *NbPirin3* expression showed a steady decline, whereas *NbPirin1*, *NbPirin4*, and *NbPirin5* expression initially declined but later demonstrated an upward trend (Figure 5A). These results suggest that *NbPirin6* is highly responsive to temperature changes and may serve a vital role in the stress adaptation of *N. benthamiana* under external temperature fluctuations. Under low temperatures (4 °C), the same five time points were assessed (Figure 5B).

These findings revealed that the expression of three *NbPirins* increased at 2 h, decreased at 4 h, and slightly increased again at 6 h. Notably, the *NbPirin2* and *NbPirin6* expression levels significantly decreased at 12 h, whereas the *NbPirin4* expression level substantially increased at this time point. Moreover, the expression of *NbPirin5* showed a continuous decline. *NbPirin3* expression showed a fluctuating but overall downwards trend. In contrast, *NbPirin1* expression almost reached its lowest level after 2 h and recovered to normal levels after 12 h.

Additionally, we selected two phytohormones, ABA and MeJA, and applied them exogenously to *N. benthamiana* for phytohormone stress treatment followed by RT-qPCR analysis. Similar to the abiotic stress experiments, we observed changes at five time points. Upon ABA treatment, the expression of *NbPirin4* steadily decreased. The expression of *NbPirin1* also showed a decreasing trend but increased at 12 h. In contrast, *NbPirin2* expression slightly increased at 4 h but was significantly decreased at all other time points. The expression of *NbPirin3* showed an overall decreasing trend, but increased at 4 h compared to 2 h, and then was significantly downregulated. The expression of *NbPirin5* showed a decreasing and then an increasing trend, recovering at 12 h to approximately the same level as at 2 h. *NbPirin6* expression showed a fluctuating but overall downwards trend (Figure 5C). Under MeJA treatment, the expression levels of *NbPirin1*, *NbPirin2*, *NbPirin3*, and *NbPirin4* initially presented significant increases. However, *NbPirin2* and *NbPirin3* expression did not sustain this increase, whereas *NbPirin1* and *NbPirin4* expression experienced a subsequent decline, followed by another increase. Notably, *NbPirin5* and *NbPirin6* did not show any upregulation under MeJA treatment but instead maintained a consistent and significant downregulation, potentially indicating a role opposite to that of other *NbPirin* members in response to MeJA (Figure 5D).

### 3.8. Expression of NbPirins During TMV, TuMV, or PVX Infection

**Figure 6:**
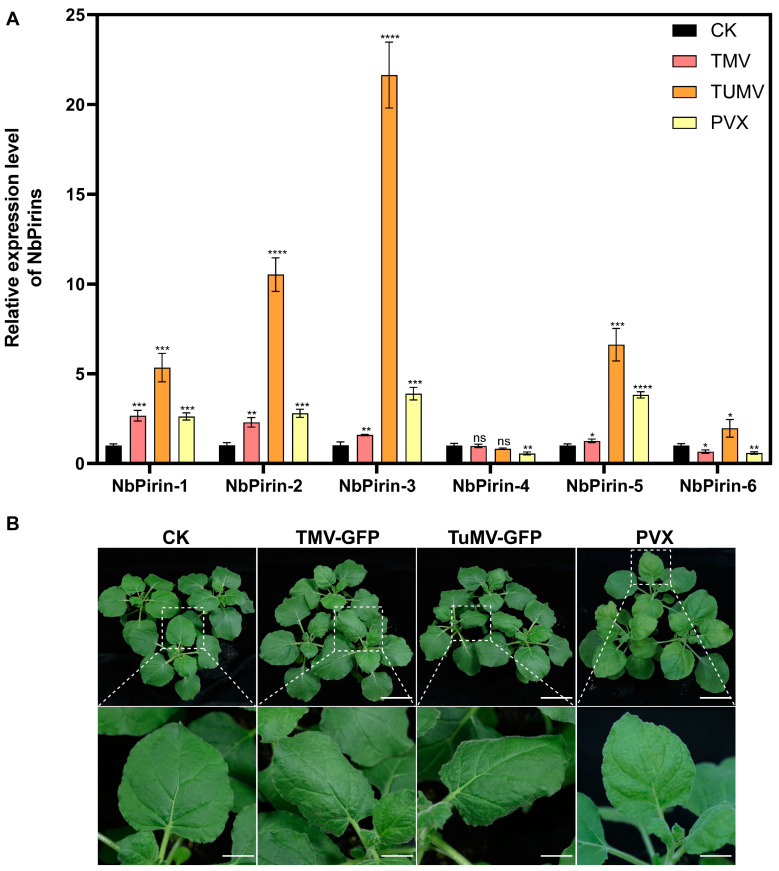
(**A**) The relative expression of *NbPirins* after viral infection (TMV, TuMV, and PVX). (**B**) The plant phenotype figure. Three independent replicates were conducted. Data is the mean ± SD (Student’s *t*-test, ns = not significant, *n* = 3, * *p* < 0.05, ** *p* < 0.01, *** *p* < 0.001, **** *p* < 0.0001).

In addition to abiotic factors, we investigated the effects of biotic stress by inoculating *N. benthamiana* with the common infecting viruses, tobacco mosaic virus (TMV), turnip mosaic virus (TuMV), and potato virus X(PVX), to explore the changes in *NbPirin* expression during viral infection. Leaves from the same positions on four-week-old *N. benthamiana* seedlings were collected seven days post-infection to examine the relative expression patterns of *NbPirins* under TMV, TuMV, and PVX infection. *NbPirin1*, *NbPirin2*, *NbPirin3*, and *NbPirin5* expression significantly increased in all viral infections. Most notably, *NbPirin3* reached a very high level of expression under TuMV infestation, and the majority of *NbPirins* reached a very high level of expression under TuMV infestation; NbPirin6 increased only under TUMV infestation, suggesting that *NbPirins* may be the key genes in *N. benthamiana* that defend against TuMV invasion. *NbPirin6* was markedly downregulated under TMV and PVX infection but was upregulated in response to TuMV. However, *NbPirin4* was either unchanged or downregulated in response to viral infection (Figure 6).

## 4. Discussion

In this study, we successfully identified six *Pirin* genes (*NbPirin1*–*NbPirin6*) in *N. benthamiana* using the Pfam database and a hidden Markov model (HMM) (Table S1). Phylogenetic analyses revealed that these *Pirin* genes were relatively conserved in other species of the Solanaceae family, such as tomatoes, peppers, and potatoes, which contain a *Pirin* superfamily domain (Figure 1A). Moreover, the six *Pirin* genes (*NbPirin1*–*NbPirin6*) could be divided into three clades (Figure 1B); this finding is consistent with the conclusions of earlier research conducted on wheat and *Arabidopsis* [7]. This implies the functional conservation of *Pirin* genes in different species. In addition, we also proved that the six *NbPirin* genes were distributed on three different chromosomes of *N. benthamiana*, with chromosome 9 showing the highest abundance (Figure 2). Further examination of the six *NbPirin* genes revealed duplicate gene pairs. We identified possible tandem repeat genes, *NbPirin5* and *NbPirin6*, with close chromosomal locations and highly similar amino acid sequences; therefore, we believe that they may have similar functions. For example, the expression of these two genes often exhibits similar trends in response to abiotic and biotic stress. This further enriches our comprehension of evolution within the *Pirin* gene family (Figure 2). When the Ka/Ks ratio was <1, the duplicate gene pair was subjected to strong purifying selection, suggesting that this duplication event was subjected to selective pressure during evolution (Table S2). Purifying selection is the process by which gene frequencies associated with a phenotypic trait are selected or eliminated when the trait no longer adapts to the current environment or breeding needs. Therefore, it is possible that some of the traits regulated by *NbPirin5* and *NbPirin6* are unfavorable for the survival of *N. benthamiana*. Therefore, functional studies on this pair of duplicated genes will help reveal the evolutionary mechanisms of the *Pirin* gene family.

It has been demonstrated that *cis*-acting elements perform a pivotal role in the regulation of diverse physiological activities in plants, including light responses, phytohormone activity, temperature regulation, and growth and development [24,37]. Consequently, we analyzed the *cis*-acting elements in the promoter region of the *NbPirin* gene. This analysis revealed the potential regulatory mechanisms of the *NbPirin* gene family in response to biotic and abiotic stress. We found that the highest proportion of elements was related to light responsiveness, indicating that *NbPirin* genes may be participating in photosynthesis and light signaling processes in plants (Figure 4). Moreover, components involved in hormone responsiveness (e.g., MeJA, SA, ABA, GA, and auxin) were abundant, indicating an important role for *NbPirin* genes in plant hormone signaling and regulation (Figure 4). In addition, the presence of components related to environmental stress (e.g., low-temperature response components, anaerobic induction-essential components, and defense-responsive components) suggests that the *NbPirin* gene family may be participating in the response and adaptation of plants to a wide range of environmental stresses. These findings provide important clues for comprehending the regulatory mechanisms and functions of the *NbPirin* gene family.

Tissue-specific analysis revealed that *NbPirin* gene expression levels differ across different tissues, which may be associated with plant growth, development, and the functions of specific tissues. For example, *NbPirin6* expression was the highest in stems, which may indicate that it is a key gene that regulates the growth and development of *N. benthamiana* leaves. However, the expression of *NbPirin5* was minimal in the leaves, and in the stems it was not expressed. This finding is analogous to the occurrence of *Ta-Pirin-5* (A, B, and D) in wheat, indicating the potential of these *Pirin* genes to perform a pivotal role in the development of the roots of *N. benthamiana* [7]. These differential expression patterns may reflect the specific functions of *NbPirin* genes in different plant tissues (Figure 5).

Abiotic stresses, such as temperature and phytohormones, have a significant impact on various aspects of plant growth and development. Plants adapt to environmental stress by regulating gene expression. We observed a significant change in the expression level of the *NbPirin* gene under high-temperature (42 °C) and low-temperature (4 °C) treatments (Figure 6A,B). The response of *NbPirin* to cold and heat stress is not completely fixed, with some of its expression being upregulated and some being downregulated, a phenomenon similar to that observed in wheat [7]. In particular, *NbPirin6* expression decreased most significantly under high-temperature treatment and again showed a very significant upregulation under low-temperature stress within 6 h, suggesting that it may play a role in adapting to external temperature stress. When plants are exposed to high-temperature stress, high-temperature signals are transmitted to cells via transduction pathways, which in turn regulate the expression of transcription factors and key heat-tolerant genes [38]. Therefore, we suspect that *NbPirin6* may also act through a similar form of regulation in plants to help *N. benthamiana* cope with external temperature stress. In addition, we conducted experiments on *N. benthamiana* related to phytohormone stress. *NbPirin3* achieved a notably elevated response level at the 2 h mark following the administration of the MeJA spray. In all experimental periods, under the influence of ABA and MeJA, *NbPirin6* was found to be significantly downregulated. This finding suggests that *NbPirin6* is a key hormone-related gene in *N. benthamiana* (Figure 5C,D). These findings suggest that *Pirin* genes perform pivotal roles in plant hormonal regulation. Previous studies have demonstrated that *Arabidopsis Pirin1* acts as an effector of other genes in different tissues and participates in different signaling mechanisms that inhibit ABA-mediated delays in seed germination [6,39]. Thus, hormonal regulation of the *NbPirin* gene may be one of its key functions in plant stress responses.

In addition to abiotic stress, biotic stress also has a significant effect on plant growth. TMV, TuMV, and PVX are important plant viruses that affect a broad spectrum of crops and cause severe yield losses [40,41,42]. Our study revealed that the expression pattern of the *NbPirin* gene changed significantly after infection with viruses such as TMV, TuMV, and PVX, and the expression of *NbPirin1*, *NbPirin2*, *NbPirin3*, and *NbPirin5* significantly increased in all viral infections. Most notably, *NbPirin3* reached a very high level of expression under TuMV infestation; combined with previous studies, we suspect that *NbPirin3* may be one of the disease resistance genes that plays the most important role in *N. benthamiana* under virus stress [43] (Figure 6). These findings indicate that *Pirin* genes may perform a pivotal role in plant antiviral immunity. For example, in barley, the transcript level of *Pirin* was increased by pathogen-derived trichothecenes, which can induce cell death [16,17]. Notably, *NbPirin6* expression was significantly downregulated in response to TMV and PVX infection, but upregulated in response to TuMV infection. *NbPirin4* was significantly downregulated only under PVX infection. The rest of the viral infections did not undergo significant changes, which is more obviously different from the situation of most *NbPirins* (Figure 6), suggesting that different viruses may regulate *Pirin* gene expression through different mechanisms. Thus, the expression patterns of *Pirin* genes may perform complex regulatory roles in the immune responses of plants to different viruses.

Although this research provides a preliminary experimental basis for the function of the *NbPirin* gene family in *N. benthamiana*, it has several limitations. For example, the specific functions of *NbPirin* genes in plant growth and development remain unclear and require further verification using techniques such as gene knockout or overexpression. In addition, the relationship between *Pirin* genes and plant disease resistance, especially the immune response to different pathogens, warrants further study. Furthermore, the interactions between *NbPirin* and other stress-related genes should be assessed through more systematic studies. To summarize, the present study revealed the diversity of the *Pirin* gene family and its expression characteristics under different stresses in *N. benthamiana* using a multi-angle experimental analysis. *Pirin* genes may perform a pivotal role in plant stress resistance, development, and immune responses, thereby providing valuable clues for further functional studies.

## 5. Conclusions

Six *NbPirin* genes were characterized in *N. benthamiana*. The *Pirins* of various Solanaceae crops (*C. annuum*, *S. lycopersicum*, *S. tuberosum*) were analyzed using a phylogenetic tree, revealing that these genes could be grouped into three distinct clades. Genome-wide analyses of gene structure, chromosomal location, domains, and *cis*-acting elements were also conducted. Furthermore, under abiotic stress (temperature and phytohormones), the expression of *NbPirin1-6* was influenced by different temperatures and phytohormones. Among these, the expression level of *NbPirin6* was notably specific, suggesting its potential role in responses to external abiotic stress. In addition, under biotic stress, we demonstrated that the expression of *NbPirin1-6* was modulated by various viral infections (TMV, TuMV, and PVX). Notably, *NbPirin3* was expressed at the highest level across multiple viral infections, highlighting its potential involvement in plant antiviral responses. In summary, this study provides a comprehensive analysis of the *Pirin* gene family in *N. benthamiana*, forming a foundation for future research on the role of *NbPirins* in plant responses to various stressors.

## Data Availability

The original contributions presented in this study are included in the article/Supplementary Materials. Further inquiries can be directed to the corresponding author.

## Supplementary Materials

The following supporting information can be downloaded at https://www.mdpi.com/article/10.3390/genes16020121/s1: Figure S1. Conserved motif and gene structure analyses. Table S1. Detailed information about six predicted Pirin proteins in *N. benthamiana*. Table S2. Ks, Ka, and Ka/Ks values calculated for paralogous *Pirin* gene pairs (*N. benthamiana*–*N. benthamiana*). Table S3. Cis-acting regulatory elements analysis of *NbPirin* gene promoters. Table S4. The primers used in this study.
