# Supplementary figures and images for: Genome-Wide Identification and Characterization of the Pirin Gene Family in Nicotiana benthamiana
Source: Genes (Basel). 2025 Jan 22;16(2):121. doi: 10.3390/genes16020121 (PMC11855294; doi:10.3390/genes16020121)

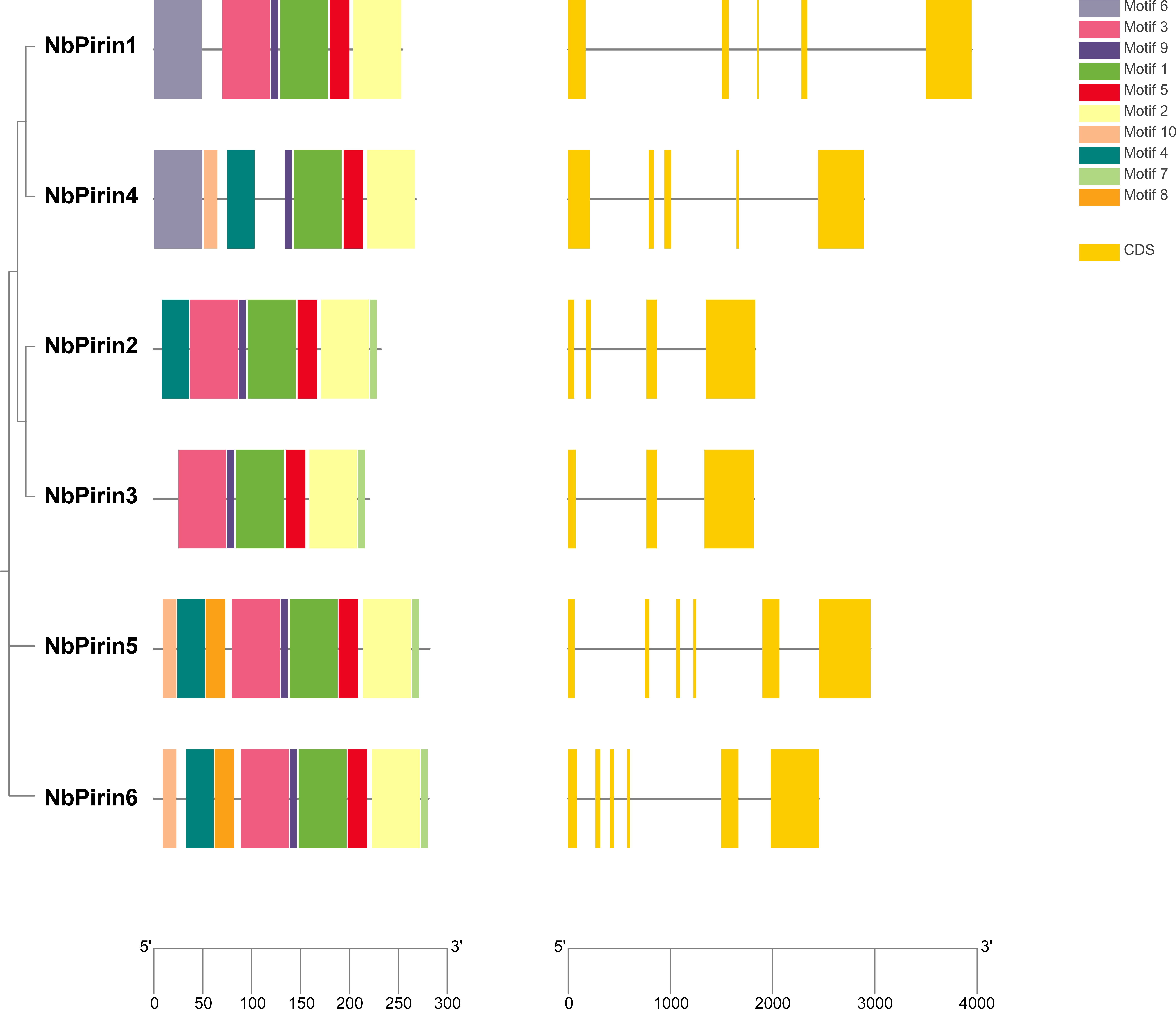

Supplement: Supplementary file 1 [file genes-16-00121-s001.zip › genes-3393782-supplementary/Figure S1. Conserved motif and gene structure analyses..jpg]
